# Reproductive Dynamics of *Sterna hirundinacea* Lesson, 1831 in Ilha dos Cardos, Santa Catarina, Brazil

**DOI:** 10.1155/2014/907549

**Published:** 2014-05-28

**Authors:** Hélio Augusto Alves Fracasso, Joaquim Olinto Branco, Márcio Amorim Efe, João Pedro Barreiros

**Affiliations:** ^1^Universidade Federal de São Carlos, CP 676, 13565-905 São Carlos, SP, Brazil; ^2^Centro de Ensino em Ciências Tecnológicas da Terra e do Mar, UNIVALI, CP 360, 88301-970, Itajaí, SC, Brazil; ^3^Setor de Biodiversidade e Ecologia, Instituto de Ciências Biológicas e da Saúde, Universidade Federal de Alagoas, Praça Afranio Jorge, s/n, Prado, 57010-020 Maceió, AL, Brazil; ^4^Azorean Biodiversity Group (CITA-A) and Platform for Enhancing Ecological Research & Sustainability (PEERS), Departamento de Ciências Agrárias, Universidade dos Açores, Rua Capitão João D'Ávila, 9700-042 Angra do Heroísmo, Portugal

## Abstract

In this work, we intend to describe the reproductive dynamics of *Sterna hirundinacea* in an island from South Brazil. We studied the reproductive biology of this species in its natural environment and provide data on their growth, survival, and reproductive success in Ilha dos Cardos, Santa Catarina, South Brazil. Samplings were carried out daily on the island throughout the reproductive seasons of 2003, 2005, and 2006 and the different stages of development of the chicks were characterized according to age, length of the beak, and plumage characteristics. We provide a basic equation Lm = 167.91 (1 − *e* ^−0.062*t*−(−0.23)^) to determine the approximate age of individuals using their body mass. The main cause of chick mortality on the island was natural (63.17% in 2003, 81.41% in 2005, and 79.96% in 2006), whereas predation contributed to mortality in a proportion of 38.83% in 2003, 18.59% in 2005, and 20.04% in 2006. The absence in the area of the chicks' main predator, Kelp gull (*Larus dominicanus*), the large number of chicks that reached the final stages of development, and their reproductive success demonstrate that Ilha dos Cardos is an important breeding site for the species in southern Brazil.

## 1. Introduction

The South American Tern,* Sterna hirundinacea* Lesson, 1831, is distributed in the Atlantic coast of South America, from southeastern Brazil to Tierra del Fuego in Argentina/Chile, including the Falkland Islands and the Pacific coast to southern Peru [1, 2], with one record for riverine environments in Rio Negro, Argentina [3].

The species is almost exclusively coastal, nesting on sandy or rocky beaches, cliffs, and small islands [4]. In the Brazilian coasts, nests are found in coastal islands from Santa Catarina [5, 6] to Espírito Santo [7], from April to August, usually in sympatry with Cabot's Tern,* Thalasseus acuflavidus* (Cabot, 1847) [6, 8, 9]. The South American Tern nests singly in scattered nests; their eggs and chicks are dark and effectively do mimics of their surroundings [10]. Chicks abandon the nests after their first week of life and seek shelter beneath neighboring vegetation [6, 8, 11].

Patterns of growth in birds are a result of evolutionary adaptations to environmental factors [12, 13] and were first qualitatively studied by describing the developmental stages in a series, followed by quantitative formulations that consider growth as a result of a network of simultaneous metabolic processes [14]. According to Nisbet et al. [15], differences in the growth patterns of chicks have been used to explore parental performance variations, whereas differences in mean parameters of colony growth over the years have been used to explore differences in average environmental conditions. It is known that the postnatal development of seabirds can be a sensitive indicator of local environmental conditions [16, 17] and has been evaluated for many species [18–21].

The breeding biology of South American Terns was described by Fracasso et al. [10], their reproductive success by Fracasso and Branco [11], and the foraging comparison with Cabot's Terns by Fracasso et al. [22]. In this study, we investigated the reproductive biology of the South American Tern in its natural environment, with the objective of giving information on growth, survival, and reproductive success of a population breeding in Cardos Island following the pioneering work of Branco [6]. Furthermore, we provide a hypothesis that, with the basic equation formed by these three-year data, we will determine the approximate age of individuals using their mass, providing more tools for the species conservation, since management of endangered bird species does require an accurate knowledge of specific breeding events such as hatching dates and growth of chicks [23].

## 2. Methods

**Figure 1:**
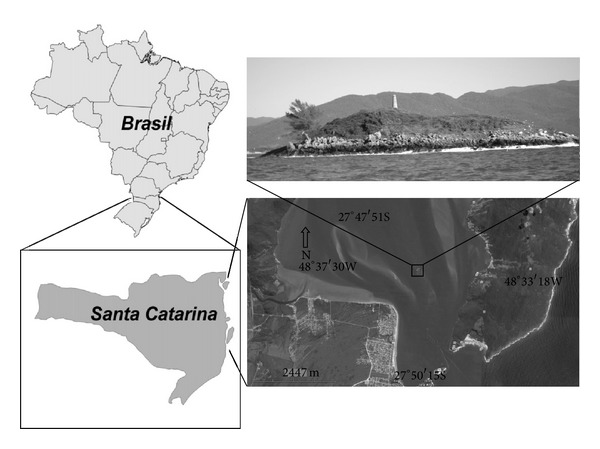
Map showing the study area [10].

Sampling was carried out daily at Ilha dos Cardos (27°48′55′′ S, 48°34′52′′ W), Florianópolis, Santa Catarina (Figure 1), during the breeding seasons of 2003, 2005, and 2006.

### 2.1. Fieldwork

According to the incidence of coupling and nests, seasons were divided into three periods: early (12/05 to 04/07), medium (05/07 to 11/08), and late (from 12/08). The chicks were sampled by direct counting according to their age. The number of dead chicks was obtained from rigorous searches conducted periodically throughout the island, when dead bodies were collected and counted. All dead chicks (regardless of the cause of death: lack of food, drowning, and territorial fights or being preyed on by hawks and vultures) were collected and disposed of to avoid counting them more than once. We followed a nest in the center of the colony, which had two eggs, daily during the 2006 season; the chicks were photographed and key changes in their plumage were recorded (Figure 16).

**Figure 2:**
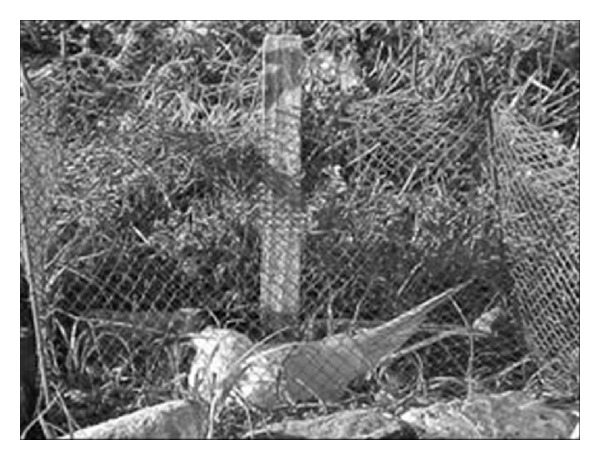
Individual fence around the nests of* Sterna hirundinacea.*

In order to monitor chicks daily, we installed individual enclosures (1 m^2^) (Figure 2) around each new posture, using fishing lines, iron hooks, and gill nets (mesh 1.0 cm), in an adaptation of the method described by Efe et al. [24].

We did this to prevent the chicks from running away and getting lost in the vicinity of the nest [23, 25]. The chicks inside and outside the enclosures were tagged and followed (Table 1) in order to conduct comparisons and analyze possible changes in growth caused by the stress of daily recapture.

Juveniles and adults were captured using mist nets as described in CEMAVE-IBAMA [26]. In this study, the measurements of the culmen (Lt beak) and tarsus (Lt tarsus) are presented in order to characterize the pattern of linear dimension of the body in the study population, and growth was mostly calculated based on the mass of the chicks. The biometrics of the culmen (Lt beak) and the length in centimeters of the tarsus (Lt tarsus) were taken using a caliper of 0.05 mm and the weight, expressed in grams (Wt), was measured using a 60, 100, 300, and 500 g PESOLA, with a precision of, respectively, 1, 2, 3, and 5 g [5].

### 2.2. Data Processing and Analyses

The Von Bertalanffy model was adjusted and adapted to the growth of juvenile terns, using the body mass, according to the formula Lm = L**∞**  (1 − *e* ^−*k*(*t*−*t*_0_)^), where Lm is the weight in grams, L**∞** is the maximum mass of juveniles, *k* is the constant of growth, *t* is the time in days, and *t*_0_ is the age at birth.

Measurements of the chicks' body structures registered in the 2003, 2005, and 2006 breeding seasons were analyzed using ANOVA, which tested for homogeneity of variance (Bartlett's test) and normal distribution (Kolmogorov-Smirnov test) [27]. When significant differences were found, the contrast of means (Tukey-Kramer) was applied to indicate which were significantly different.

## 3. Results

### 3.1. Age Characterization

**Figure 3:**
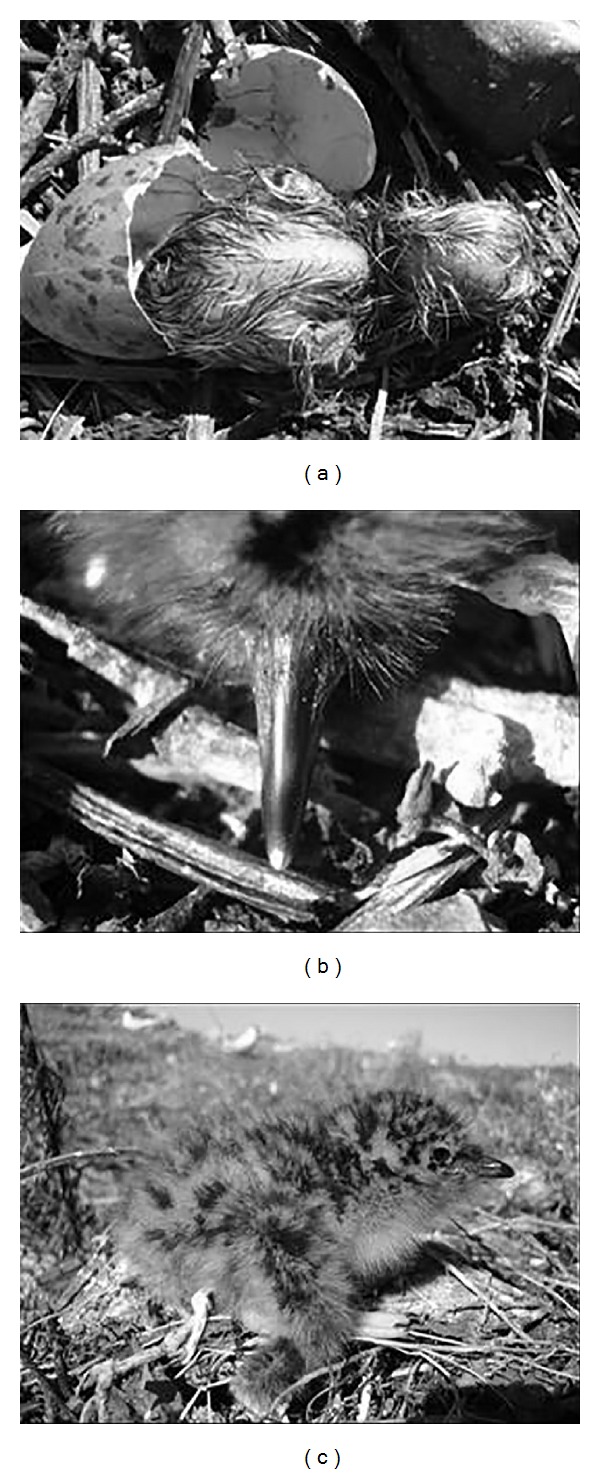
Beginning of the JI phase of* Sterna hirundinacea* (a), egg tooth detail (b), and color of the plumage and end of the stage (c).

The different stages of development of the chicks were established according to their age, length of the beak culmen (Figure 10), and plumage characteristics. Thus, Juvenile I (JI) corresponded to the hatchlings usually found in the nest, with the “egg tooth” (structure at the nozzle tip used to break the egg from the inside) (Figure 3) and Lt_beak_  average 1,23 ± 0.01 cm between 1 and 6 days, with yellow-cream plumage throughout the body (Figure 3).

**Figure 4:**
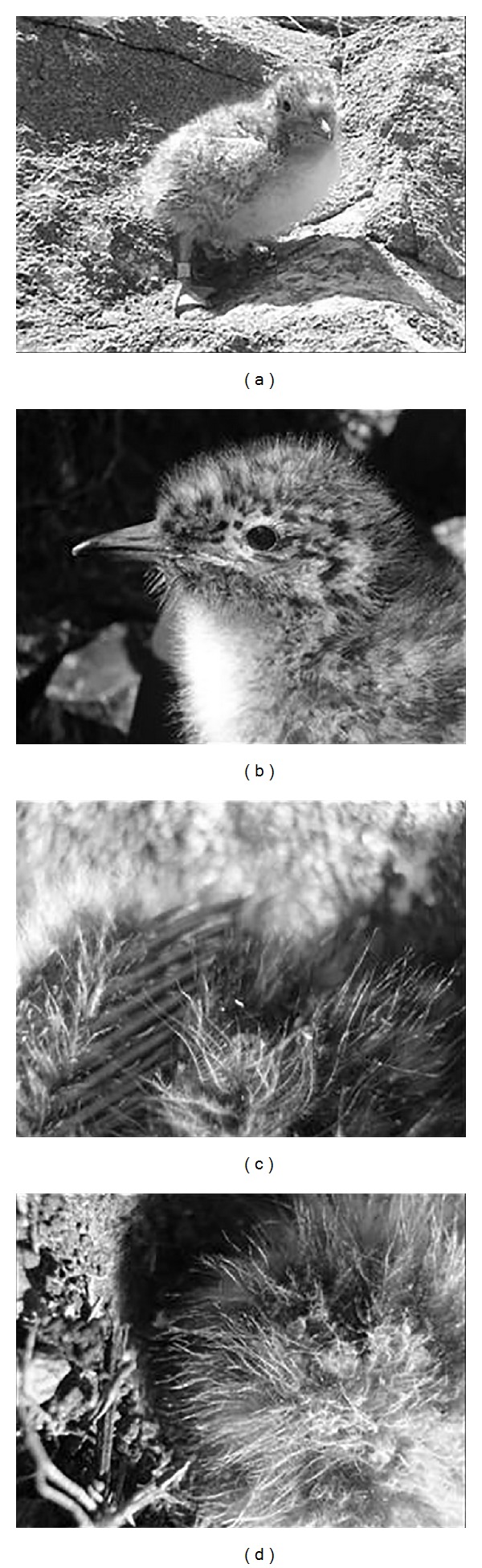
JII of* Sterna hirundinacea* (a), details of the beak without the egg tooth (b), details of the remiges (c), and rectrices (d).

Juvenile II (JII) corresponded to individuals without the “egg tooth” (Figure 4), tarsus thick enough for the final metal tag, 7 to 14 days old, Lt_beak_  average 1.70 ± 0, 02, with white breast and yellow-cream plumage on the rest of the body (Figure 4).

**Figure 5:**
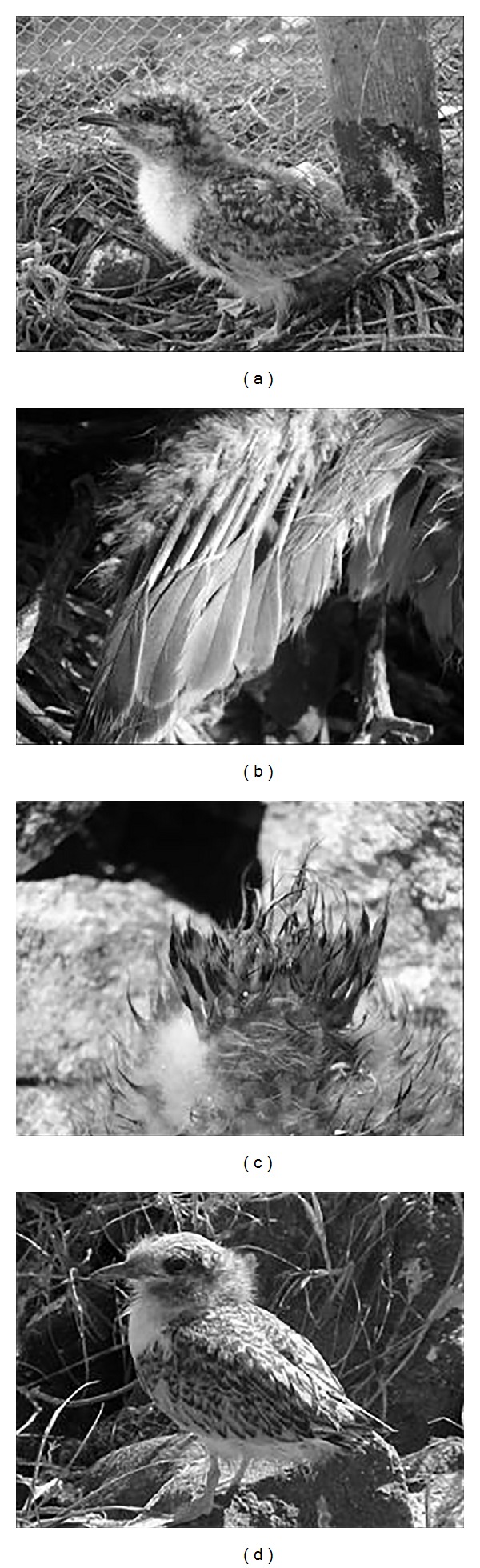
JIII of* Sterna hirundinacea* in the beginning of this phase (a), details of the cannon feathers on the remiges (b), rectrices (c), and end of the stage (d).

Juvenile III (JIII) corresponded to 15–28-day-old individuals and average Lt_beak_  2.21 ± 0.01, cannon feathers on the remiges (Figure 5), and some cream-yellow plumage on the head (Figure 5).

**Figure 6:**
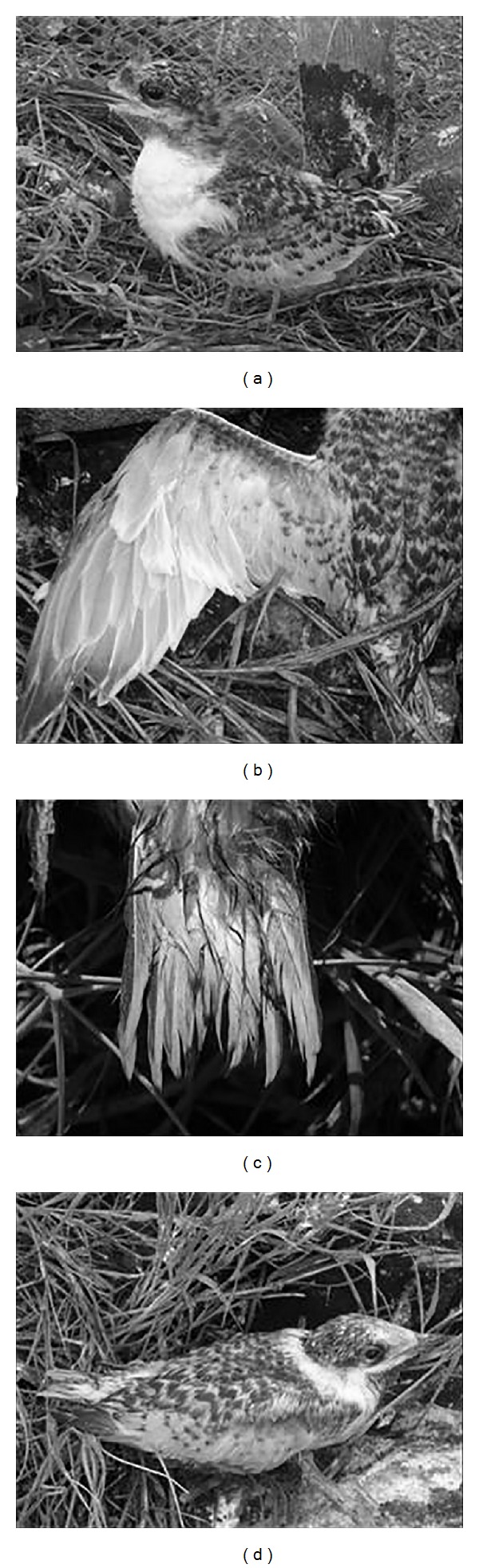
JIV of* Sterna hirundinacea* in the beginning of the phase (a), details of the cannon feathers on the remiges (b), rectrices (c), and end of the stage (d).

Juvenile IV (JIV) corresponded to chicks with well-developed retrix feathers, Lt_beak_  mean 2.65 ± 0.03, without yellow-cream plumage, and aggressive behavior, 29 to 37 days old (Figure 6).

**Figure 7:**
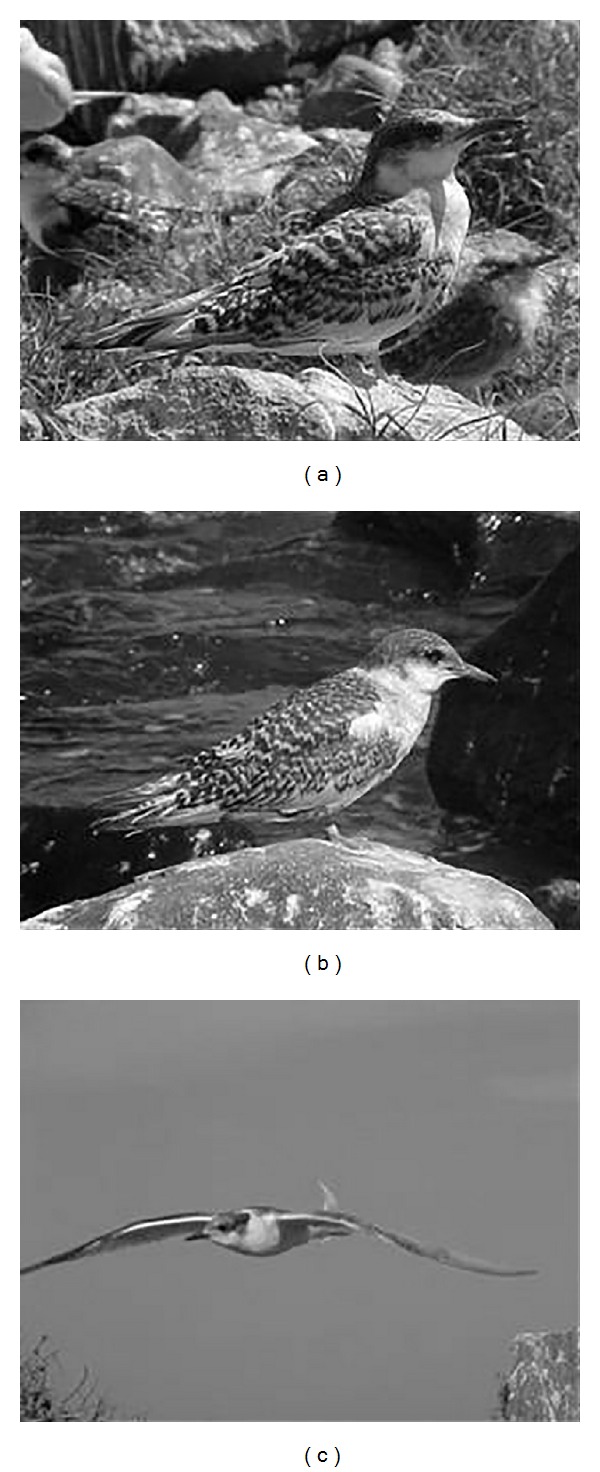
*Sterna hirundinacea* juvenile perched (a) and (b) and in flight (c).

Juveniles (Jv) had mean Lt_beak_  2.83 ± 0,02 cm, were 38 days old or older, had no trace of the chick plumage, and were already able to fly (Figure 7).

### 3.2. Age Group Distribution

In 2003, the first eggs hatched in July, with a large increase in the numbers of JI until mid-July (15/07, *n* = 525), followed by a sharp fall; this trend in fluctuating daily abundance was recorded for all subsequent ages, with the greatest numbers of JII in late July (26/07, *n* = 413), JIII in early August (03/08, *n* = 325), and JIV in mid-August (12/08, *n* = 274); the young began to emerge in late July (29/07), about 38 days after the first outbreaks, with the greatest abundance in late August (24/08, *n* = 265) and a gradual decrease until the abandonment of the colony.

In 2005, there were three outbreak peaks, the first and largest at the end of June (21/06, *n* = 590), the second in early August (11/08, *n* = 54), and the last, and smallest, in mid-September (14/09, *n* = 26). The peak abundance of other ages was observed in early July (JII: 05/07, *n* = 475 and JIII: 10/07, *n* = 362) and late July (JIV: 22/07, *n* = 320 and Jv: 31/07, *n* = 225), respectively.

In 2006, the first chick was recorded in late May (31/05), with a gradual increase of JI until the end of June (20/06, *n* = 485), followed by a steady decrease in their numbers until the absence of neonates at the end of the reproductive period. Still early in the season, in mid-June, the numbers of JII were the greatest (29/06, *n* = 412), whereas peaks in the numbers of JIII (09/07, *n* = 358) and JIV (17/07, *n* = 358) occurred in mid-July. According to the same figure, the number of individuals in the juvenile phase fluctuated from the end of July (24/07, *n* = 287) to mid-August (19/08, *n* = 152), with gradual abandonment of the colony by the end of September.

### 3.3. Chicks' Mortality

**Figure 8:**
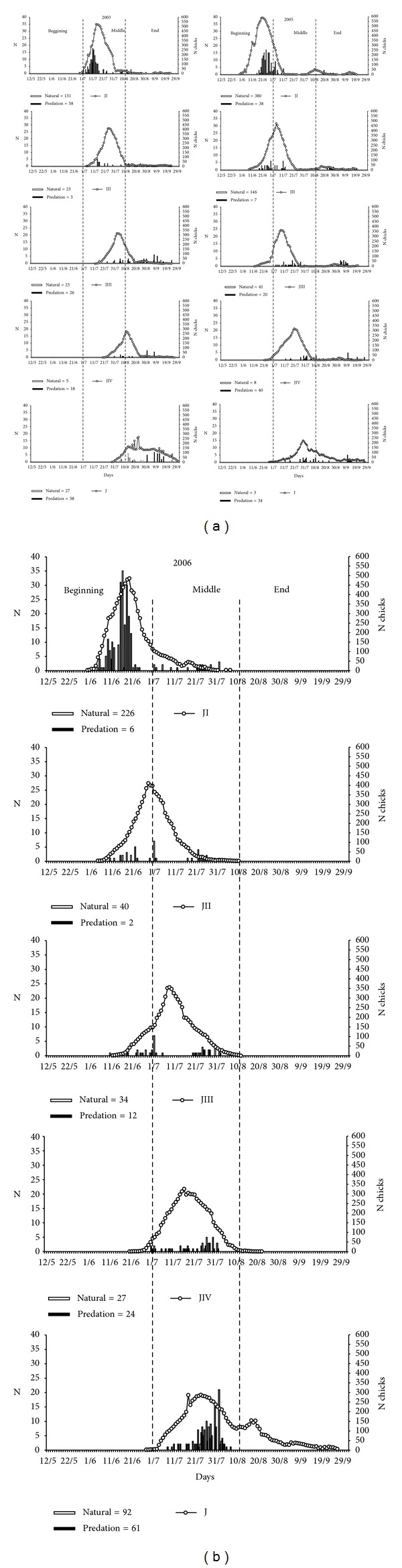
Abundance, mortality by natural causes, and daily predation of young of* Sterna hirundinacea* at Ilha dos Cardos during the reproductive phases of 2003, 2005, and 2006.

The main cause of chicks' mortality at Cardos Island was natural, accounting for 63.17% of deaths in 2003, 81.41% in 2005, and 79.96% in 2006, whereas predation contributed to 38.83%, 18.59%, and 20.04%, respectively (Figure 8).

In 2003, mortality from natural causes affected mainly young JI and JII in the middle of the reproductive period; predation, on the other hand, was more intense when chicks reached JIII, from the middle to the end of the breeding season. In 2005, natural causes were more prevalent at the beginning of the season, killing JIs and JIIs, and in the middle and end of it, killing JIIIs. Predation, by contrast, was the main mortality factor for JIVs and Jvs at the end of the breeding season. In 2006, mortality from natural causes was a result of fights for territory early in the season (on younger chicks) and lack of food at the end (JIII, JIV, and Jv), along with predation of older individuals in the middle of the reproductive period.

### 3.4. Growth

**Figure 9:**
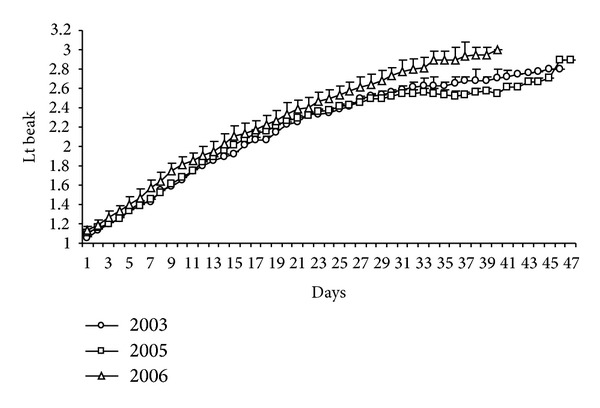
Daily increment in the growth (cm) of the beak of* Sterna hirundinacea.*

**Figure 10:**
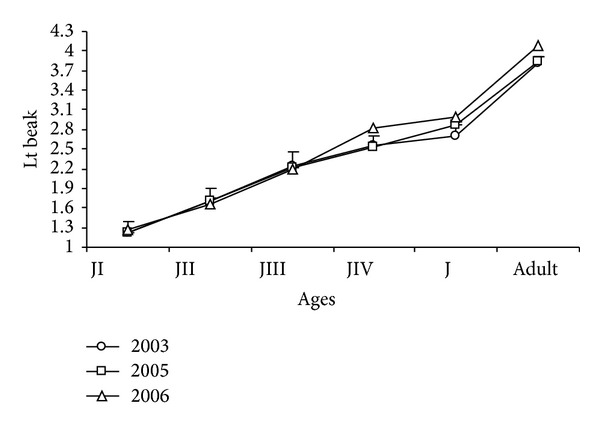
Mean length of the beak (cm) of* Sterna hirundinacea* chicks.

During the breeding seasons, an increase in the size of the beak was observed throughout the development, but these did not reach the adult size while individuals stayed in the colony. There were no differences in the size of the beak of chicks up to 23 days in the three years of study. However, in 2006, growth rates were higher and chicks remained on site for a fewer number of days (Figure 9).

Mean beak measurements were the following for each developmental state, as defined in this work: JI 1.23 ± 0.01 cm, JII 1.70 ± 0.02, JII 2.21 ± 0.01, JIV 2.65 ± 0.03, Jv 2.83 ± 0.02, and adults 3.99 ± 0.04 cm.

The ANOVA test did not detect significant differences in beak measurements during the study for age groups JII (*F*_2-826_ = 0.72; *P* > 0.05) and JIII (*F*_2-1354_ = 2.53; *P* > 0.05). Significantly higher values, by contrast, were found for JI (*F*_2-1190_ = 7.69; *P* < 0.001), JIV (*F*_2-869_ = 6.56; *P* < 0.05), Jv (*F*_2-95_ = 57.11; *P* < 0,001), and adults (*F*_2-14_ = 6.77; *P* < 0.05) in 2006.

**Figure 11:**
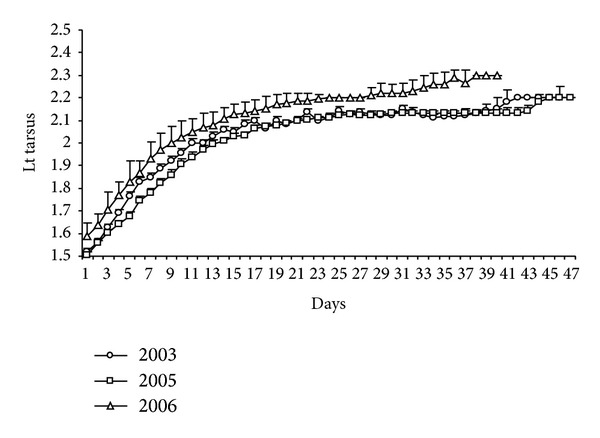
Daily growth of the tarsus (cm) of* Sterna hirundinacea* chicks.

The greatest daily increase in structure size was observed for the tarsus until day 17; after that, tarsal length increased slowly up to day 38 (Figure 11).

The highest growth rate for this structure was then observed from age group JI (1.64 ± 0.01 cm) to JII (1.97 ± 0.02 cm), with small increases in JIII (2.11 ± 0.01 cm) and JIV (2.15 ± 0, 01 cm). The tarsus of juveniles resembled (2.20 ± 0.01 cm) the means observed for the adults (2.26 ± 0,02 cm).

**Figure 12:**
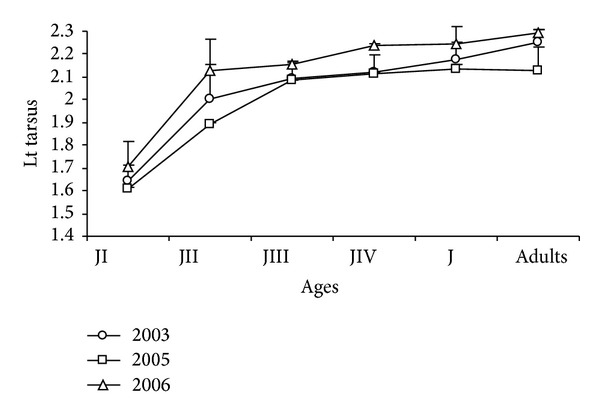
Mean of the tarsus (cm) of* Sterna hirundinacea* chicks.

According to the results of the ANOVA, significant differences in the size of the tarsus were observed at all ages, with the greatest values found in 2006, the year responsible for most variation in JI (*F*_2-1190_ = 30.82; *P* < 0.001), JIII (*F*_2-1354_ = 92.69; *P* < 0.001), JIV (*F*_2-869_ = 156.10; *P* < 0.001), Jv (*F*_2-95_ = 4.98; *P* < 0.005), and adults (*F*_2-14_ = 7.36; *P* < 0.05). The smallest variation was observed in 2005 for JII (*F*_2-825_ = 7.91; *P* < 0.001) (Figure 12).

**Figure 13:**
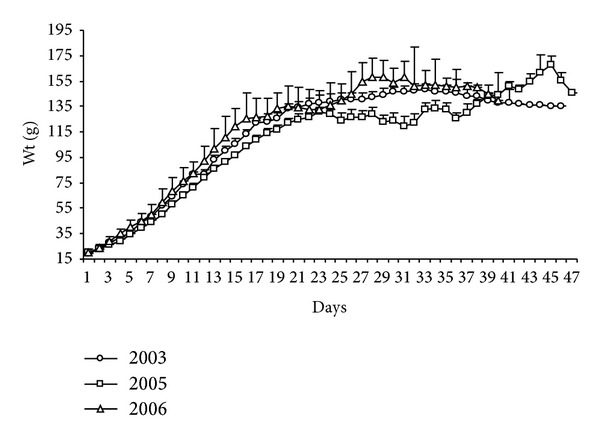
Daily increase in the weight (g) of* Sterna hirundinacea* chicks.

The daily gain in mass from the first to the 20th day of life was considerable for* S. hirundinacea* chicks at Ilha dos Cardos (19.00 to 115.00 g), with a small increase in the curve until day 37 (143.42 g), when the chicks started to take short flights at the edge of the colony, thereby losing some weight (Figure 13).

**Figure 14:**
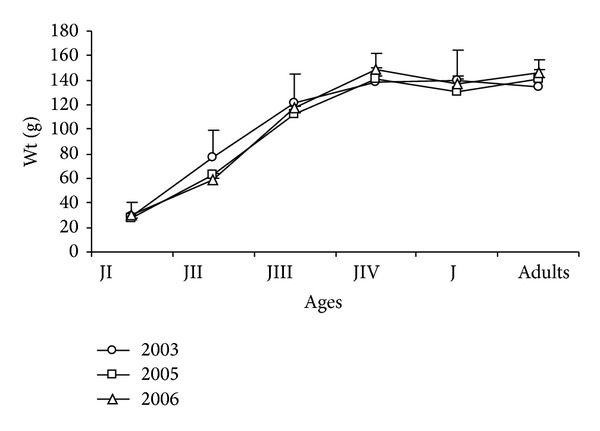
Mean weight (g) of* Sterna hirundinacea* chicks.

In all three stages, chick development was high, from 28.26 ± 0.30 g to JII (67.34 ± 0.71) and JIII (116.23 ± 0.64 g), with a small increase for JIV (143.95 ± 0.69) and a decrease in the Jv phase (137.97 ± 2.69 g) and a tendency for the growth curve to stabilize once adulthood was reached (142.82 ± 6.82 g) (Figure 14).

Weight varied significantly, according to the ANOVA test, between age groups JI (*F*_2-1190_= 7.35; *P* < 0,001) and JIII (*F*_2-1354_ = 18.23; *P* < 0,001), reaching the lowest values in 2005, and varied for JII (*F*_2-826_ = 68.55; *P* < 0,001), JIV (*F*_2-869_= 13.54; *P* < 0,001), and adults (*F*_2-14_ = 9.89; *P* < 0,001), particularly in 2003 and 2006, being not significant for Jv (*F*_2-95_ = 0.35; *P* > 0.05).

Chicks in age group JII, which were randomly captured in the colony during 2003, had significantly bigger beaks and more mass than those which remained cloistered; however, the mass of cloistered JIII, JIV, and Jv was greater than of those who remained free at the site (Table 2).

In 2005, we recorded significant differences in beak measurements at age groups JI, JIII, and JIV, caused by the values found for free chicks, whereas the variation in the size of the tarsus was more significant in the younger age group JI and less in groups JIII and JIV. Variation in weight was greater in age groups JI and JIII (Table 2). According to the same table, JI chicks found free in 2006 had significantly larger dimensions in the structures measured than chicks that remained enclosed, with the exception of body mass recorded at age groups JIII, JIV, and Jv.

**Figure 15:**
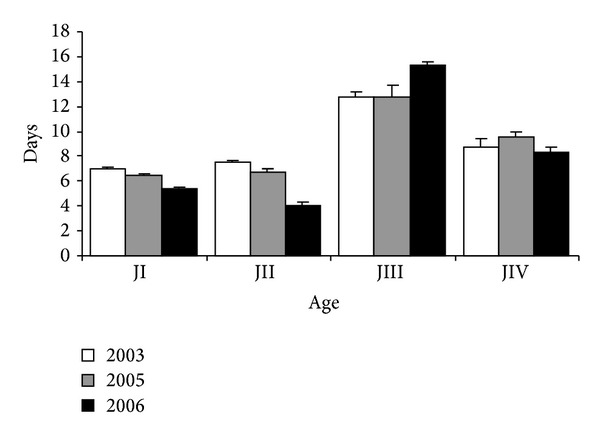
Mean time and days necessary for* Sterna hirundinacea* chicks to reach ages I, II, III, and IV.

It took on average 6.48 ± 0.11 days for the chicks to lose their “egg tooth” and to be classified in group JII (*n* = 151) (Figure 15). Up to the appearance of feathers on the remiges and rectrices, with consequent classification in age group JIII (*n* = 128), it took on average 6.55 ± 0.20 days. From age group JIII to JIV, characterized by the development of the remiges and rectrices and complete loss of primary feathers on the head, it took longer (13.33 ± 0.38 days, *N* = 103); learning to fly going from age group JIV to Jv took on average 8.67 ± 0.43 days (*N* = 21) (Figure 15.)

When the breeding seasons were compared, significant statistical differences were observed in the time required to outgrow age groups JI (*F*_2-184_ = 16.65; *P* < 0.001), JII (*F*_2-125_ = 28.36; *P* < 0.001), and JIII (*F*_2-100_ = 4.13; *P* < 0.05) but not JIV (*F*_2-18_ = 0.30, *P* > 0.05).

**Figure 16:**
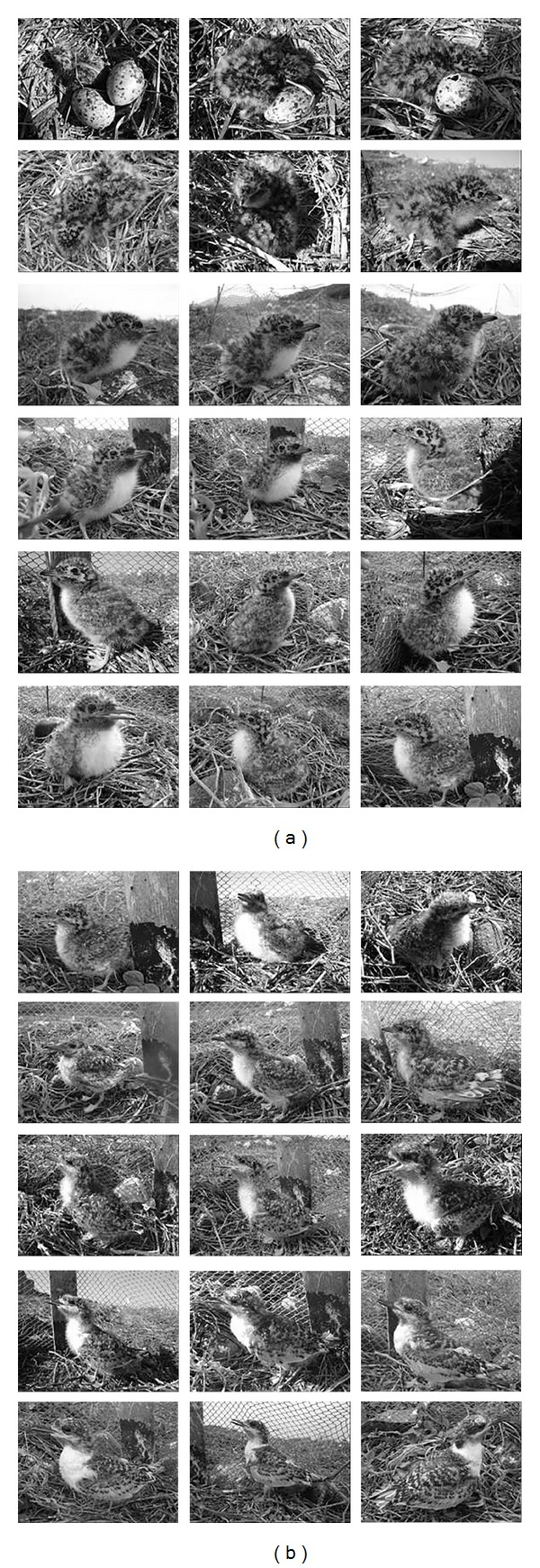
Daily transformation of a* Sterna hirundinacea* chick.

The second chick broke at three days after the first; however, it did not survive after the ninth day of nest monitoring, being alive for only six days. The cause of death was malnutrition and competition with its older brother (Figure 16). The first chick grew up alone, reaching the JII stage in six days, JIII in seven days, and JIV in 27 days. After 33 days, with its feathers well developed the chick was able to perform small jumps, temporarily abandoning the fence, and was observed in the nest in the following days, in the presence of its parents (Figure 16).

**Figure 17:**
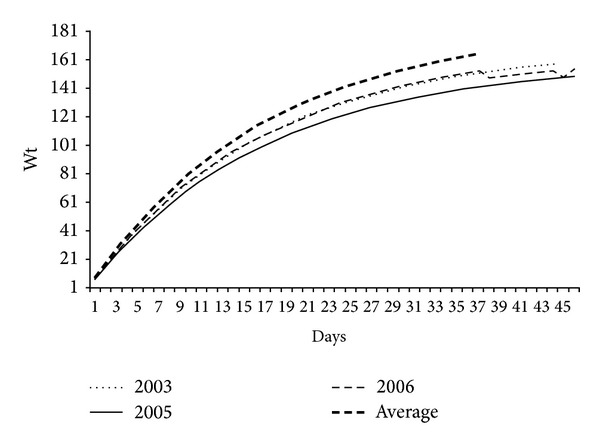
Model of adjustment of the growth curve of* Sterna hirundinacea* chicks at Ilha dos Cardos during the breading seasons of 2003, 2005, and 2006.

The Von Bertalanffy growth model for the mass of the young of* S. hirundinacea* that were surrounded and captured at random, at the Ilha dos Cardos, fit the following equations: Lm = 170.35  (1 − *e* ^−0.061*t*−(−0.18)^) in 2003, Lm = 159.14  (1 − *e* ^−0.061*t*−(−0.18)^) in 2005, Lm = 185.84  (1 − *e* ^−0.060*t*−(−0.23)^) in 2006 (Figure 17), and Lm = 167.91  (1 − *e* ^−0.062*t*−(−0.23)^) among the three years.

The fastest rate of growth occurred in the first 20 days of life, with small additions until the flight and the consequent abandonment of the island, approximately on the 46th day in 2003, 45th in 2005, and 42nd in 2006 (Figure 17).

## 4. Discussion

### 4.1. Age Group Distribution

The postnatal development of seabird chicks can be a sensitive indicator of local environmental conditions [16, 17, 28–30], which makes the use of seabirds to monitor marine environments a feasible practice [29, 30].

*Sterna hirundinacea* chicks at Ilha dos Cardos, in this study, and in other islands of Santa Catarina, leave the nest after the first two days of life and seek shelter beneath the surrounding vegetation [6] similar to the description by Nisbet [31] for* S. hirundo* (Linnaeus, 1758).

In colonies of* S. hirundinacea*, the interval between the birth of the first to the second chick was 53.8 hours, and parental care varied between 26 and 29 days [32], whereas, in Ilha dos Cardos, the time elapsed between the onset of the first to the second chick ranged between 48 and 60 hours, and the parental care interval, from hatching to the chicks' first flight, was a little longer, approximately 35–41 days, which may be a result of different reproductive phenologies (austral winter for the Brazilian populations and austral summer for the Argentine population) and the various influences of different oceanographic factors (Brazil and Malvinas currents, resp.). Local adaptations influenced directly by the climate affect the availability of seasonal food, and important factors for the growth and development of the offspring import in the cost of foraging [33], thereby influencing the reproductive success [34]. Also, metabolism increases with the latitude [35].

### 4.2. Chicks' Mortality

In the coast of Espírito Santo, adult* S. hirundinacea* arise in mid-April and the first chicks begin to emerge in mid-June [8]. In Ilha dos Cardos, chicks were born between 20 and 25 May, and there was a gradual increase in the number of births beginning in June and peaking in September [6]. In the following season (present study), chicks began to emerge in early July with the greatest abundance in August. This variation is considered normal in birds [36] and has to do with the environmental conditions encountered by the birds in the beginning of the breeding season.

At Ilha dos Cardos, chicks' mortality is mainly from natural causes. Constant storms during the winter in southern Brazil had been previously recognized as important for other colonies of terns in Brazil [37, 38]. In Argentina, the main causes of death were predation and parental desertion or delays to return to the nest [32]. The absence of* L. dominicanus* (Lichtenstein, 1823) populations reproducing in Ilha dos Cardos contributed to the low threat of predation, since this predator is the biggest threat to the conservation of terns in Brazil and Argentina [38, 39].

The most critical period in the survival of tern chicks is during the first 10 days of their lives [40–43] and the probability of survival [44] and the growth curve [45] of the chicks also show that there is a critical period during the first nine days after the eclosion in the Ilha dos Cardos.

In colonies of the species in Argentina, the chicks' risk of death decreases, until they acquire a different plumage, between 13 and 17 days of life and from 26 to 28 days of life [32]. The same happens in breeding colonies of* T. acuflavidus* in Espírito Santo [21], where the biometric parameters of the studied chicks do not reach the adult size before the preflight period.

### 4.3. Growth Curve and Chick Ageing

Being a semiprecocious and migratory bird,* S. hirundinacea* develops rapidly, which is reflected in an accelerated growth rate of the tarsus in the initial stages, when compared with other structures. According to Klaassen [35], in the species of terns studied by him, the tarsus develops fast and reaches about 90% of its final length midway between birth and recruitment, indicating that all are able to move about. Tarsus and body mass grew more quickly than culmen, attaining asymptotic values at about 14 and 16 days, respectively, for Whiskered Tern* Chlidonias* hybrid at Lake Grand-Lieu (47°05′ N, 1°39′ W) in western France [23]. This rapid initial growth may be advantageous for allowing the chick to leave the nest within a few days and move from the colony in search of shelter and protection from predators.

According to Branco [6],* S. hirundinacea* JI individuals in Ilha Itacolomi and Ilha dos Cardos were not significantly different in terms of average mass, reaching up to 30 g, whereas JIIs from Ilha dos Cardos were significantly heavier. When the chicks reached the JIII stage, however, those from Ilha de Itacolomi were heavier. In this season, according to our data, the mass of the offspring from Ilha dos Cardos followed the same trend until JIII from other years but was larger at JIV and Jv phase.

The relationship between body mass and length of the culmen in* S. hirundinacea* individuals from Itacolomi and Cardos indicates that the species has negative allometric growth (Wt = 27.5450 Lt^1.84908490^); consider *r* ^2^ = 0.8583, with the greatest increases in mass occurring in the initial length classes [6]. The growth curve of Argentina's population adjusted close to the logistic equation (*r* ^2^ = 0.98) [32].

The growth curves of* S. hirundo* and* S. dougallii* (Montagu, 1813) consist of an exponential phase (initial and short), followed by a linear and logarithmic growth, which tends to an asymptotic value at 19 days for the first species and 24 days for the second [46]. The same trend was observed for the growth curve of daily gain in weight of chicks of* S. hirundinacea* of Ilha dos Cardos, which stabilized later, around the 25th day of life. Nisbet et al. [42, 43] also found that growth predicted chick survival accurately in* S. dougallii*. In that species, survival could be predicted from body mass growth in the first few days after hatching. In contrast, in* T. sandvicensis *(Latham, 1787) body mass in the first few days after hatching is not important for survival, suggesting that poor nourishment during an early stage of life could be overcome [25]. Nisbet et al. [42] suggest that chick growth and survival, which are already manifested during the first days after hatching in Roseate Terns, are primarily determined by parental performance. In Sandwich Terns, it seems relatively easy for most parents to meet the food requirements of their newly hatched chicks [25].

The probability of survival [44] and the growth curve [45] of chicks indicate that there is a critical period during the first nine days after hatching, an aspect also referred to by Hogan et al. [47] while studying a breeding colony in an islet (Deserta) that is near our own study site. The main causes of death in Argentina were predation (4.8%) and parental desertion or delays to return to the nest (11.1%). The risk of death decreases until the chick acquires another plumage, when it is between 13 and 17 days old [32]. According to the same author, the risk of death increases again when the chicks are between 26 and 28 days of life, and the main causes of death were parental desertion and changes in the frequency of visits to the chicks (8.3%). Hogan et al. [47] also report predation by* L. dominicanus* on both eggs and chick as the main cause of reproductive failure in* S. hirundinacea*. The largest mass was observed in individuals of age groups JIII, JIV, and Jv which remained fenced in. The lower energy expenditure and the absence of displacement around the colony may have been responsible for it. The smallest mass was found in JII individuals that remained cloistered and may have been influenced by use the fence, while suggesting the need to remove it after the critical period of the chicks' survival. The growth curve equation established in this study can help researchers identify the age of individuals based on their mass. The fact that* L. dominicanus* does not reproduce in the area, the great amount of chicks reaching adulthood, and the reproductive success demonstrated in the breeding seasons analyzed make Ilha dos Cardos an important breeding site of the species in southern Brazil.

## Figures and Tables

**Table 1:** *Sterna hirundinacea* chicks captured and monitored during the 2003, 2005, and 2006 breeding seasons.

Age	2003		2005		2006	
	Cloistered	Free	Cloistered	Free	Cloistered	Free
JI	440	170	474	25	206	19
JII	206	258	373	28	206	18
JIII	241	119	502	109	366	26
JIV	118	90	288	205	161	33
Jv	10	25	2	4	18	21

Total	1015	662	1639	371	860	117

**Table 2:** Mean size and standard deviation of beak and tarsus length and weight of cloistered and free chicks at Ilha dos Cardos during the 2003, 2005, and 2006 breeding seasons.

Age	Est	2003			2005			2006		
		Cloistered	Free	ANOVA	Cloistered	Free	ANOVA	Cloistered	Free	ANOVA
JI	Lt b	1.23 ± 0.01	1.23 ± 0.02	*F*_(1.608)_ = 0.005 (*P* > 0.05)	1.22 ± 0.01	1.29 ± 0.04	*F*_(1.497)_ = 6.47 (*P* < 0.05)	1.25 ± 0.01	1.42 ± 0.05	*F*_(1.221)_ = 32.70 (*P* < 0.001)
	Lt t	1.65 ± 0.01	1.66 ± 0.01	*F*_(1.608)_ = 0.487 (*P* > 0.05)	1.60 ± 0.01	1.70 ± 0.04	*F*_(1.497)_ = 13.31 (*P* < 0.001)	1.68 ± 0.01	1.92 ± 0.06	*F*_(1.221)_ = 59.52 (*P* < 0.001)
	Wt	30.19 ± 0.67	29.30 ± 0.67	*F*_(1.608)_ = 0.504 (*P* > 0.05)	26.74 ± 0.44	29.88 ± 2.67	*F*_(1.497)_ = 2.43 (*P* < 0.05)	28.10 ± 0.58	43.34 ± 4.01	*F*_(1.221)_ = 49.96 (*P* < 0.001)

JII	Lt b	1.66 ± 0.02	1.72 ± 0.01	*F*_(1.462)_ = 6.059 (*P* < 0.05)	1.71 ± 0.04	1.75 ± 0.05	*F*_(1.399)_ = 0.068 (*P* > 0.05)	1.63 ± 0.01	1.77 ± 0.03	*F*_(1.125)_ = 16.48 (*P* < 0.001)
	Lt t	1.99 ± 0.01	2.02 ± 0.01	*F*_(1.462)_ = 3.254 (*P* > 0.05)	1.89 ± 0.01	1.92 ± 0.05	*F*_(1.399)_ = 1.111 (*P* > 0.05)	2.12 ± 0.05	2.15 ± 0.05	*F*_(1.125)_ = 0.005 (*P* > 0.05)
	Wt	74.78 ± 1.64	80.51 ± 1.52	*F*_(1.462)_ = 6.508 (*P* < 0.05)	62.68 ± 0.92	59.95 ± 3.53	*F*_(1.399)_ = 0.619 (*P* > 0.05)	58.68 ± 1.15	59.75 ± 4.41	*F*_(1.125)_ = 0.102 (*P* > 0.05)

JIII	Lt b	2.21 ± 0.02	2.26 ± 0.02	*F*_(1.358)_ = 3.551 (*P* > 0.05)	2.20 ± 0.01	2.27 ± 0.02	*F*_(1.609)_ = 20.37 (*P* < 0.001)	2.19 ± 0.01	2.22 ± 0.03	*F*_(1.390)_ = 0.284 (*P* > 0.05)
	Lt t	2.13 ± 0.01	2.15 ± 0.02	*F*_(1.358)_ = 1.734 (*P* > 0.05)	2.09 ± 0.01	2.06 ± 0.01	*F*_(1.609)_ = 11.77 (*P* < 0.001)	2.15 ± 0.01	2.19 ± 0.01	*F*_(1.390)_ = 4.395 (*P* < 0.05)
	Wt	124.42 ± 1.57	114.91 ± 2.10	*F*_(1.358)_ = 12.62 (*P* < 0.001)	109.69 ± 0.99	123.79 ± 2.28	*F*_(1.609)_ = 36.07 (*P* < 0.001)	119.94 ± 1.20	90.62 ± 3.28	*F*_(1.390)_ = 40.92 (*P* < 0.001)

JIV	Lt b	2.57 ± 0.01	2.61 ± 0.01	*F*_(1.206)_ = 3.167 (*P* > 0.05)	2.59 ± 0.01	2.63 ± 0.01	*F*_(1.491)_ = 10.05 (*P* < 0.01)	2.85 ± 0.15	2.73 ± 0.02	*F*_(1.192)_ = 0.124 (*P* > 0.05)
	Lt t	2.23 ± 0.02	2.20 ± 0.02	*F*_(1.206)_ = 1.864 (*P* > 0.05)	2.13 ± 0.01	2.12 ± 0.01	*F*_(1.491)_ = 3.942 (*P* < 0.05)	2.24 ± 0.01	2.23 ± 0.03	*F*_(1.192)_ = 0.687 (*P* > 0.05)
	Wt	147.19 ± 2.04	130.31 ± 2.36	*F*_(1.206)_ = 29.35 (*P* < 0.001)	143.92 ± 1.06	145.13 ± 1.32	*F*_(1.491)_ = 0.728 (*P* > 0.05)	154.57 ± 1.12	120.21 ± 4.56	*F*_(1.192)_ = 114.5 (*P* < 0.001)

J	Lt b	2.67 ± 0.02	2.64 ± 0.03	*F*_(1.33)_ = 0.401 (*P* > 0.05)	2.80 ± 0.01	2.90 ± 0.04	*F*_(1.4)_ = 1.333 (*P* > 0.05)	3.00 ± 0.02	2.99 ± 0.02	*F*_(1.37)_ = 0.102 (*P* > 0.05)
	Lt t	2.14 ± 0.03	2.15 ± 0.03	*F*_(1.33)_ = 0.073 (*P* > 0.05)	2.10 ± 0.01	2.15 ± 0.03	*F*_(1.4)_ = 1.333 (*P* > 0.05)	2.26 ± 0.01	2.23 ± 0.01	*F*_(1.37)_ = 3.595 (*P* > 0.05)
	Wt	139.10 ± 5.46	123.80 ± 3.76	*F*_(1.33)_ = 4.939 (*P* < 0.05)	137.00 ± 12.00	127.25 ± 18.76	*F*_(1.4)_ = 0.112 (*P* > 0.05)	148.89 ± 6.45	126.76 ± 5.58	*F*_(1.37)_ = 6.808 (*P* < 0.05)
